# A New Promising Material for Biological Applications: Multilevel Physical Modification of AgNP-Decorated PEEK

**DOI:** 10.3390/nano13243079

**Published:** 2023-12-05

**Authors:** Jana Pryjmaková, Daniel Grossberger, Anna Kutová, Barbora Vokatá, Miroslav Šlouf, Petr Slepička, Jakub Siegel

**Affiliations:** 1 Department of Solid-State Engineering, University of Chemistry and Technology Prague, 166 28 Prague, Czech Republic; jana.pryjmakova@vscht.cz (J.P.); dan.grossberger@seznam.cz (D.G.); kutovaa@vscht.cz (A.K.); jakub.siegel@vscht.cz (J.S.); 2 Department of Microbiology, University of Chemistry and Technology Prague, 166 28 Prague, Czech Republic; barbora.vokata@vscht.cz; 3 Institute of Macromolecular Chemistry, Academy of Sciences of the Czech Republic, Heyrovského nám. 2, 162 06 Prague, Czech Republic; slouf@imc.cas.cz

**Keywords:** polyetheretherketone, immobilisation, physical modification, nanostructures, antibacterial properties, biocompatibility

## Abstract

In the case of polymer medical devices, the surface design plays a crucial role in the contact with human tissue. The use of AgNPs as antibacterial agents is well known; however, there is still more to be investigated about their anchoring into the polymer surface. This study describes the changes in the surface morphology and behaviour in the biological environment of polyetheretherketone (PEEK) with immobilised AgNPs after different surface modifications. The initial composites were prepared by immobilising silver nanoparticles from a colloid solution in the upper surface layers of polyetheretherketone (PEEK). The prepared samples (Ag/PEEK) had a planar morphology and were further modified with a KrF laser, a GaN laser, and an Ar plasma. The samples were studied using the AFM method to visualise changes in surface morphology and obtain information on the height of the structures and other surface parameters. A comparative analysis of the nanoparticles and polymers was performed using FEG-SEM. The chemical composition of the surface of the samples and optical activity were studied using XPS and UV-Vis spectroscopy. Finally, drop plate antibacterial and cytotoxicity tests were performed to determine the role of Ag nanoparticles after modification and suitability of the surface, which are important for the use of the resulting composite in biomedical applications.

## 1. Introduction

In recent years, the field of biomedical engineering has witnessed remarkable advances in material science, particularly in the development of novel surface modification techniques to enhance the functionality and performance of biomedical devices. One such material that has gained significant attention is polyetheretherketone (PEEK). With its excellent mechanical properties, biocompatibility, and resistance to harsh environments, PEEK has emerged as a promising candidate for various biomedical applications [1,2,3]. However, to further expand its potential in the healthcare sector, researchers have been exploring strategies to improve the antibacterial properties of PEEK, as microbial colonisation remains a persistent challenge in medical devices.

The anchoring of AgNPs to the polymer surface can be achieved through various methods, including chemical reduction, physical deposition, or in situ preparation [4,5,6,7]. One effective method for anchoring AgNPs is to immobilise them into the polymer near the surface with an excimer laser. This kind of composite represents a long-term source of Ag^+^ ions [8]. Once AgNPs are successfully immobilised, surface modification techniques are employed to further enhance the material’s properties. These techniques can include plasma treatment, laser ablation, chemical functionalisation, or a combination of these approaches.

Plasma treatment is a widely used surface modification technique that involves subjecting the PEEK surface to a low-temperature plasma. This process modifies the chemistry and topography of the surface, resulting in improved wettability and increased surface energy [9]. Plasma treatment can also introduce functional groups that facilitate the attachment of biomolecules and promote cell interactions, making the modified surface more biocompatible. Another effective surface modification method is laser ablation, which involves the use of high-energy lasers, such as KrF excimer lasers, to remove material from the PEEK surface. Laser ablation creates nanostructures and increases surface area, providing an ideal substrate for the immobilisation of AgNPs [8,10]. This technique allows precise control over the surface morphology and can be tailored to specific applications, resulting in improved integration with surrounding tissues. Furthermore, the combination of nanometals and plasma or laser treatment represents a synergistic effect on antibacterial activity [11,12,13,14,15,16].

The combination of AgNP immobilisation and subsequent surface modification holds great potential for a wide range of biomedical applications. By leveraging the unique properties of AgNPs and the benefits of surface modification techniques, researchers aim to develop PEEK-based materials with superior antibacterial capabilities, reduced risk of infections, and enhanced biocompatibility [17,18,19,20,21]. These advances will pave the way for the design and fabrication of biomedical devices, such as implants, catheters, and prosthetics, that offer improved performance and patient outcomes.

In this paper, we explore the recent advances in surface modification techniques following AgNP immobilisation on PEEK. We go into the underlying principles of each technique, their resulting effects on surface morphology, and their improvements in antibacterial properties and biocompatibility. Furthermore, we discuss the potential applications of these modified surfaces in various biomedical fields, highlighting their significance in combating bacterial colonisation and advancing the development of safer and more effective medical devices.

## 2. Materials and Methods

### 2.1. General Materials

Silver electrodes (5.0 × 1.1 × 0.2 cm^3^) with a purity of 99.95% used for the electrochemical synthesis of AgNPs were obtained from Safina a.s., Vestec, Czech Republic. As stabiliser, a sodium citrate dihydrate (Na_3_C_6_H_5_O_7_, Sigma–Aldrich Co., St. Louis, MO, USA) was used. Immobilisation was carried out in a foil of polyetheretherketone (PEEK, thickness 50 µm, density 1.3 g·cm^−3^, Goodfellow Cambridge Ltd., Huntingdon, UK). *Staphylococcus aureus* (*S. aureus*; CCM 4516) and *Escherichia coli* (*E. coli*; CCM 4517) were obtained from the Czech Collection of Microorganisms, Bohunice, Czech Republic, and primary lung fibroblasts (MRC-5) were obtained from the American Tissue Culture Collection, Manassas, VA, USA. Other biological agents such as Minimal Essential Medium Eagle (MEM) and L-glutamine (a stable dipeptide) were purchased from Sigma–Aldrich, St. Louis, MO, USA.

### 2.2. Synthesis of AgNPs

Colloid silver nanoparticles (AgNPs) were prepared by electrochemical synthesis. Two silver electrodes were immersed perpendicularly to each other with a distance of 5 cm between them in sodium citrate dihydrate solution (volume 120 mL, concentration 1 mmol·L^−1^) and powered by a DC power supply (voltage 15 V, PS-305D). Synthesis was carried out for 20 min under vigorous magnetic stirring (750 r·min^−1^, IKA C-MAG HS 7, Sigma–Aldrich Co., USA). Subsequently, 100 mL of colloid solution was decanted and filtered in an Erlenmeyer flask and kept in a dark place for 24 h. The next day, the concentration of the AgNP solution was determined using atomic absorption spectroscopy and diluted to a working concentration of 30 mg·L^−1^.

### 2.3. Preparation of Samples

AgNPs were immobilised into a polyetheretherketone surface (PEEK) with polarised monochromatic light (wavelength 248 nm, UV-grade fused silica, model PBSO-248-100) from the KrF excimer laser (COMPex PRO 50 F, Coherent Inc., Santa Clara, CA, USA). The PEEK foil was placed in a spectroscopic cuvette (Starna Scientific Ltd., Ilford, UK, type 3/Q/100) filled with AgNP solution. The cuvette was then placed in a sample holder perpendicular to the laser radiation. The parameters were set as follows: 6000 pulses, pulse duration 20–40 ns, frequency 10 Hz, laser fluence 10 mJ·cm^−2^, aperture 5 × 10 mm^2^. The immobilisation was carried out with the aim of preserving the planar surface. Further details of the adopted methodology can be found in ref. [22], where the complete mechanism of immobilisation was described.

The prepared samples with immobilised nanoparticles (Ag/PEEK) were irradiated with a KrF excimer laser and GaN laser. A group of samples (Ag/PEEK/KrF7, Ag/PEEK/KrF13) was irradiated with a KrF excimer laser under the same conditions as described above with laser fluence of 7 and 13 mJ·cm^−2^. The second group (Ag/PEEK/GaN60, Ag/PEEK/GaN240) was irradiated with a GaN laser (wavelength 405 nm, objective 50×, zoom 6×), which was part of the Olympus LEXT OLS3100 confocal laser scanning microscope (Olympus Corporation, Tokyo, Japan). The exposure times were 60 and 240 s. These samples had a limitation for some analyses, as the modified area was 30 × 50 µm.

The other method for surface modification was plasma treatment, which was performed using a sputter coater, model SCD 050, BAL-TEC, set in etching mode. The conditions of the experiment were: pressure 4–6 Pa, gas argon, discharge power 8 W, electrode distance 50 mm. The samples were exposed to plasma for 60 and 240 s and are denoted as Ag/PEEK/P60 and Ag/PEEK/P240 in the text.

### 2.4. Analytical Methods

To determine the concentration of synthesised nanoparticles, atomic absorption spectrometry (AAS) was used. Measurements were made with the Agilent 280FS AA flame atomiser spectrometer (Agilent Technologies, Tokyo, Japan). The measurement error was 4%.

Atomic force microscopy (AFM) was used to study the surface morphology of Ag/PEEK samples after individual modifications. Measurements were made on a Dimension ICON device, Bruker Corp., Billerica, MA, USA, using the so-called ScanAsyst^®^ tapping mode in air. The analysis was carried out with a SANASYST-AIR probe with a silicon tip and a SiN cantilever (Bruker Corp., USA) with an elasticity constant of 0.4 N·m^−1^ and a natural frequency of 70 kHz. Data were acquired at a scan rate of 0.5 Hz and evaluated using the NanoScope^®^ analysis programme (Bruker Corp., Billerica, MA, USA). This step also obtained information on the mean surface roughness (*R*_a_), the periodicity of the structures (*L*), and the value of the surface area difference (SAD) parameter, which expresses the ratio of the difference between the measured and scanned area to the scanned area. Periodicity was measured at five different positions and arithmetical means and standard deviations were calculated.

The visualisation of the surface and arrangement of AgNPs was accomplished with the MAIA 3 field emission gun scanning electron microscope (FEG-SEM), TESCAN, Brno, Czech Republic. Before analysis, samples were fixed with double-sided carbon tape (Cristine Groepl, Tulln, Austria) on a brass stub and provided with a carbon layer in a JEE-4C vaporiser (JEOL, Akishima, Japan). Visualisation took place in high-resolution mode with a secondary electron detector at an accelerating voltage of the primary electron beam of 3 kV.

Information about the chemical composition of the samples was obtained using X-ray photoelectron spectroscopy (XPS). The samples were analysed in the ESCAProbeP Omicron Nanotechnology spectrometer (Scienta Omicron, Taunusstein, Germany) with a monochromatic X-ray source with an energy of 1486.7 eV and a pressure of 2 × 10^−8^ Pa at a take-off angle of 90° (perpendicularly). Data were processed as a graph.

Because silver nanoparticles were used, the optical activity of the prepared samples was studied using UV-Vis spectroscopy. Absorption was measured on the Lambda 25 spectrometer (Perkin Elmer, Waltham, MA, USA) in the range of wavelengths from 350 to 700 nm with the scanning rate set at 240 nm⋅min^−1^. The absorption spectra were obtained using the PerkinElmer UV WinLab v4.2 software.

### 2.5. Biological Tests

An antibacterial activity study was carried out on Ag/PEEK, Ag/PEEK/KrF7, and Ag/PEEK/KrF13 using drop plate tests estimated by counting viable bacteria [23]. In this work, two bacterial strains were chosen: the Gram-positive bacteria (G+) *Staphylococcus aureus* (*S. aureus*; CCM 4516) and the Gram-negative bacteria (G−) *Escherichia coli* (*E. coli*; CCM 4517). First, the inoculum was prepared using one colony of each bacterial strain into 4 mL (*S. aureus*) or 25 mL (*E. coli*) of Luria–Bertani (LB) liquid medium. The inocula were incubated overnight on an orbital shaker at 37 °C. The next day, they were diluted in phosphate-buffered saline (PBS) at a final concentration of approximately 12 × 10^3^ (*S. aureus*) and 2 × 10^3^ (*E. coli*) per ml. The samples were then immersed in 2 mL of bacterial suspension in test tubes. After 3 and 24 h, five drops (25 μL) from each tube were dripped onto plate count agar (PCA). The culture was carried out overnight at 37 °C. The next day, colony-forming units (CFUs) were counted, and the arithmetical means and standard deviations were calculated. Data were processed as a graph with error bars. All experiments were carried out under sterile conditions.

For the cytotoxicity tests, Ag/PEEK, Ag/PEEK/KrF7, and Ag/PEEK/KrF13 were chosen as samples with very different surface morphologies. Their cytotoxic effects were observed in primary lung fibroblasts (MRC-5,) incubated in Minimal Essential Medium Eagle (MEM) supplemented with 2 mM L-glutamine under standard conditions (37 °C, 5% CO_2_) using a resazurin assay [24]. The experimental setup was the same as that from our study dealing with Au nanowires [25]. Relative cell viability was represented by the ratio of metabolically active cells to metabolically active control cells, expressed as a percentage. The mean values and standard deviations were calculated and processed in graphs as error bars.

## 3. Results and Discussion

### 3.1. Surface Characterisation

**Figure 1:**
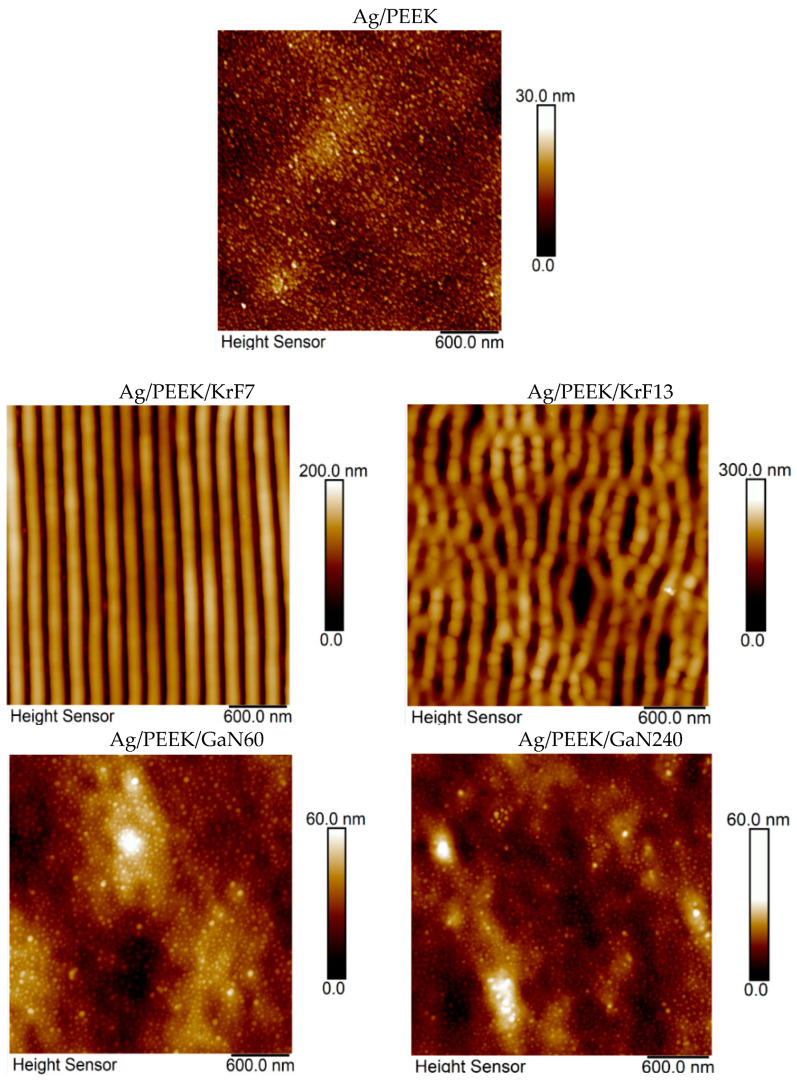
Images of the Ag/PEEK surface after KrF laser irradiation with 7 (Ag/PEEK/KrF7) and 13 mJ·cm^−2^ (Ag/PEEK/KrF13) and after GaN laser irradiation for 60 (Ag/PEEK/GaN60) and 240 s (Ag/PEEK/GaN240) obtained using AFM.

Changes in the surface morphologies of Ag/PEEK/KrF7 and Ag/PEEK/KrF13 after modification with a KrF laser and of Ag/PEEK/GaN60 and Ag/PEEK/GaN240 after modification with a GaN laser were studied. Scans with visible changes in surface morphology are shown in Figure 1 and adequate surface parameters such as *R*_a_, *L*, and *SAD* are summarised in Table 1. In Figure 1 (Ag/PEEK), it is obvious that the immobilisation of AgNPs in the near-surface layer of PEEK was successful and the planar surface was preserved as expected [22]. Subsequent modification with the KrF laser dramatically changed the surface morphology. On the surface of Ag/PEEK/KrF7 and Ag/PEEK/KrF13, nanostructures occurred. Nanostructures formed on the polymer substrate are known as LIPSS (laser-induced polymer surface structures), which can be arranged in ripples or in globular structures [10,25]. These structures arise after polarised laser irradiation of polymer substrates, which can absorb UV radiation due to their structures [26].

The laser fluence of 7 mJ⋅cm^−2^ caused periodically organized ripples with a periodicity of 210 nm. In the case of Ag/PEEK/KrF13, the ripples started to degrade and they transformed into globular structures, which are typically seen with higher laser fluence [27]. As LIPSS formed, the *R*_a_ reached about 30 nm and the SAD increased significantly. However, AgNPs were hidden under the nanostructures. Although the KrF excimer laser created nanostructures on the surface of Ag/PEEK, irradiation with the GaN laser led to material transport; however, this occurred without self-assembling, as the condition for nanostructure formation is polarised radiation [28]. After a 60 s irradiation time, the surface was seeded with larger nanoparticles; after 240 s, the small nanoparticles became more visible, and most of the large ones were gone. This phenomenon may be related to the size-dependent distribution of AgNPs throughout the polymer surface [22]. The mass transfer of the polymer gradually uncovers each layer of immobilised nanoparticles, which is correlated with the values of *R*_a_ and SAD. 

**Figure 2:**
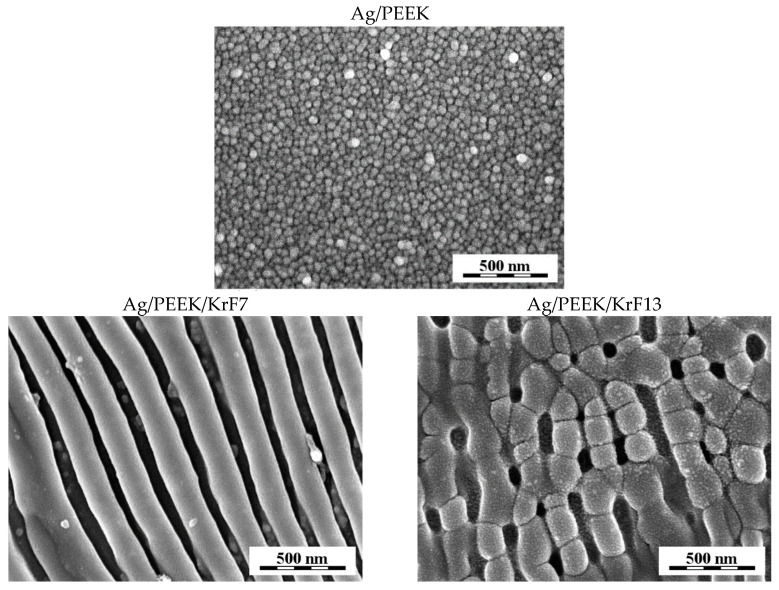
Micrographs of Ag/PEEK, Ag/PEEK/KrF7, and Ag/PEEK/KrF13 depicting nanostructures formed after KrF laser irradiation.

**Figure 3:**
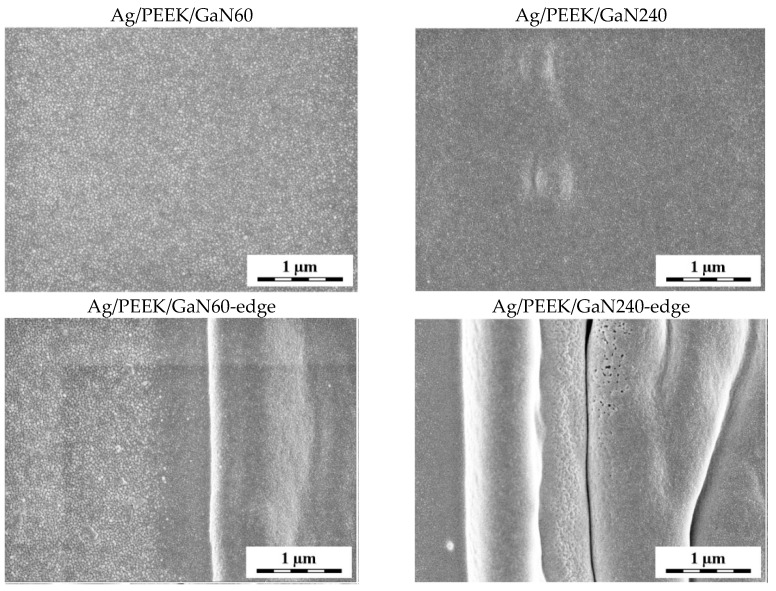
Micrographs of Ag/PEEK/GaN60 and Ag/PEEK/GaN240 with visible AgNPs and additional images of the interface between the unmodified and modified surface of Ag/PEEK, noted as Ag/PEEK/GaN60-edge and Ag/PEEK/GaN240-edge.

**Figure 4:**
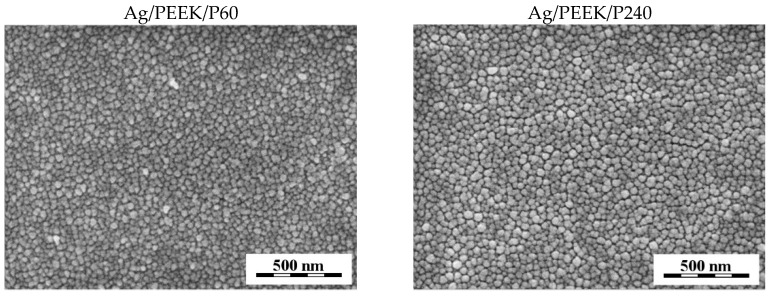
Micrographs of Ag/PEEK/P60 and Ag/PEEK/P240 after exposure to Ar plasma.

To understand the processes that take place on the polymer surface, the samples were visualised using scanning electron microscopy. Images of Ag/PEEK after each modification are shown in Figure 2, Figure 3 and Figure 4, where white to light grey represent AgNPs and darker shades of grey represent the polymer substrate. The control samples of Ag/PEEK had homogeneously distributed AgNPs on the surface, which is in agreement with the results obtained using AFM. After KrF laser irradiation, the polymer nanostructures occurred without nanoparticles. The nanoparticles that are hidden under the structures are visible in Figure 2 (Ag/PEEK/KrF7). However, the smallest AgNPs were transferred to the surface of the nanostructures after laser irradiation with 13 mJ·cm^−2^ (Ag/PEEK/KrF13). 

In Figure 3, it can be seen that surface modification with the GaN laser exposed nanoparticles that are immobilised deeper in the polymer surface. The surface of the Ag/PEEK/GaN60 sample contained AgNPs of various sizes, while the surface of Ag/PEEK/GaN240 was covered with small nanoparticles. On closer observation, it is possible to notice the dark colouration of the background around the nanoparticles (represents polymer), which means that the irradiation penetrated deeper into the composite with increasing irradiation time. In that case, the content of AgNPs decreased, and the polymer content increased. Similar results were found in the study by Elashnikov et al. [29], which deals with the irradiation of Ag-doped PMMA fibres with a laser of 405 nm wavelength.

As LIPSS did not form in this case, the wrinkling of the material occurred at the edge of the modified area, which is shown in Figure 3 (Ag/PEEK/GaN60-edge, Ag/PEEK/GaN240-edge). The wrinkling was probably caused by the photoactivation of AgNPs, as the wavelength of 405 nm is close to their LPSR [11,30]. The UV irradiation of AgNPs can heat them to 130 °C. This has an effect on the transfer of polymer material as the temperature is close to the PEEK glass transition temperature [22,31]. However, ablation of the material may not be excluded. 

Here, we obtain the first results of Ag/PEEK after the Ar plasma modification. Figure 4 contains micrographs of the Ag/PEEK/P60 and Ag/PEEK/P240 surfaces. The analysis did not show any significant differences between Ag/PEEK and Ag/PEEK/P60. Interestingly, for Ag/PEEK/P240, visible space between nanoparticles was observed, which could be explained by oxidation or material ablation. In summary, these results show that PEEK is the most susceptible to KrF laser modification in terms of surface morphology.

**Figure 5:**
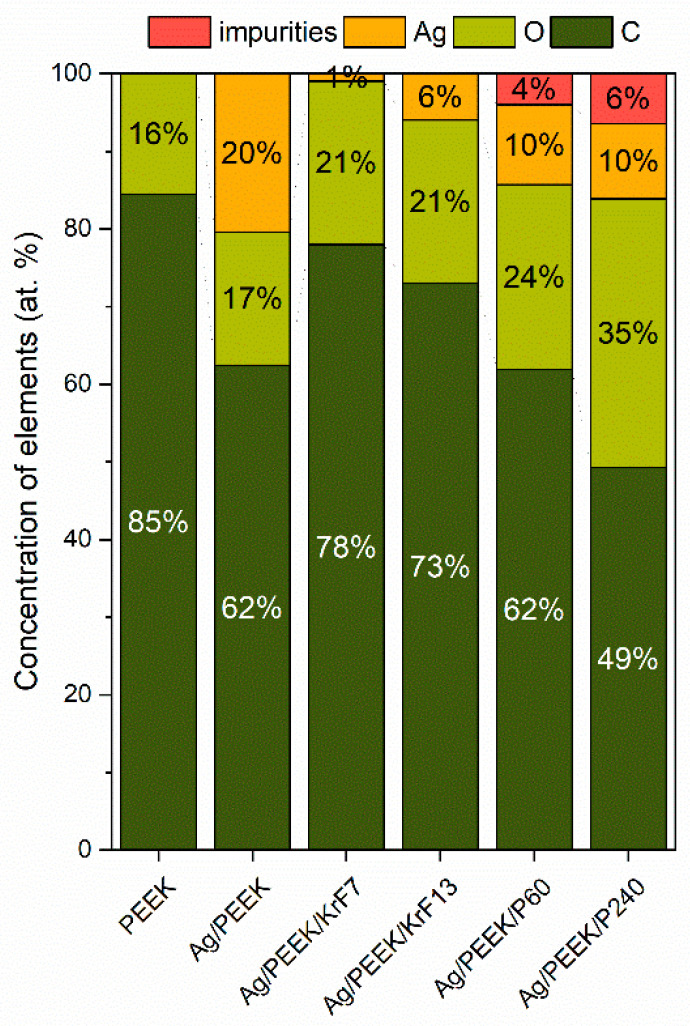
Graph showing the concentrations of carbon (C 1s), oxygen (O 1s), and silver (Ag 3d) on the surface of the Ag/PEEK composite and its modified versions.

To detect any changes in surface chemistry, the concentrations of elements in the surfaces of Ag/PEEK, Ag/PEEK/KrF7, Ag/PEEK/KrF13, Ag/PEEK/P60, and Ag/PEEK/P240 were determined, which are summarised in Figure 5. PEEK was used as a pristine control. The XPS, which can obtain information at a depth of 20 nm [32], confirmed the presence of AgNPs in the surface as the take-off angle was 90°. Surface irradiation with the KrF excimer laser significantly decreased the silver content from 20 to 6%; in the case of 7 mJ·cm^−2^, it was even lower. The decrease was caused by the creation of LIPSS that completely cover the nanoparticles. As we expected from the FEG-SEM analysis, nanoparticles occurred in a small amount on the surface of the LIPSS after a higher laser fluence. On the other hand, the concentration of oxygen slightly increased. The interaction of laser irradiation and polymer leads to many complex processes; one of them is the partial oxidation of the polymer [33]. 

The plasma-modified surface brings about more interesting results. Although the surface morphologies of Ag/PEEK, Ag/PEEK/P60, and Ag/PEEK/P240 looked pretty similar, the chemistries of these surfaces were different. The concentration of silver was reduced by half of its original value. Plasma treatment led to oxidation of the polymer, which is also evidenced by increased oxygen with prolonged treatment [34]. However, it is not possible to say with certainty whether only the polymer or the nanoparticles were oxidised. We expected that nanoparticles would not provide any changes in chemical composition as Ar plasma was used, regardless of the presence of 4–6% impurities and the possibility of nanoparticle oxidation. The chamber was cleaned prior to each modification; however, impurities such as iron and silicon were still present. The source of iron and silicon could be the sputter coater itself, as most of the components were made of iron alloys and the cylinders were made of silicon glass. Moreover, these residues could be the cause of limitations in the AFM measurement.

**Figure 6:**
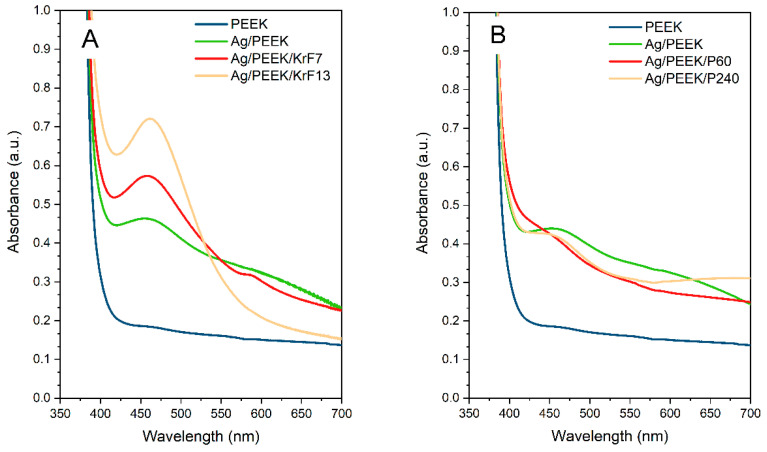
UV-Vis spectra of composites after (**A**) KrF laser irradiation and (**B**) Ar plasma treatment.

The UV-Vis spectra of the chosen samples are shown in Figure 6. Since the controls did not show optical activity, all samples that contained silver nanoparticles exhibited an optical absorption of about 450 nm. Similar trends were reported by Zeng et al. in their work on the silver–aromatic polymer composite AgNPs/PS (polystyrene) and AgNPs/AP (acrylonitrile-polystyrene), where the absorption band was 450–460 nm [35]. As can be seen in Figure 6A, composites irradiated with the KrF laser show a decreasing trend of absorbance. The peaks became narrower and more distinct with increasing laser fluence. This phenomenon can be explained by differences in the behaviour of the nanoparticles. In the case of Ag/PEEK, the silver nanoparticles appeared as a continuous layer. The layer of silver nanostructures that created it is known to have a specific absorption spectrum depending on its thickness or the conditions of preparation [36,37]. With increasing laser fluence, the nanoparticles became more separated (see Figure 2) such that their optical response was close to that of colloid nanoparticles. The results of the plasma-treated samples show the opposite trend of optical absorbance. After modification for 60 s, the concentration of silver dramatically decreased, which was reflected in a non-committal peak at 445 nm. Nonetheless, prolonged plasma treatment did not produce any dramatic changes.

### 3.2. Antibacterial Tests

**Figure 7:**
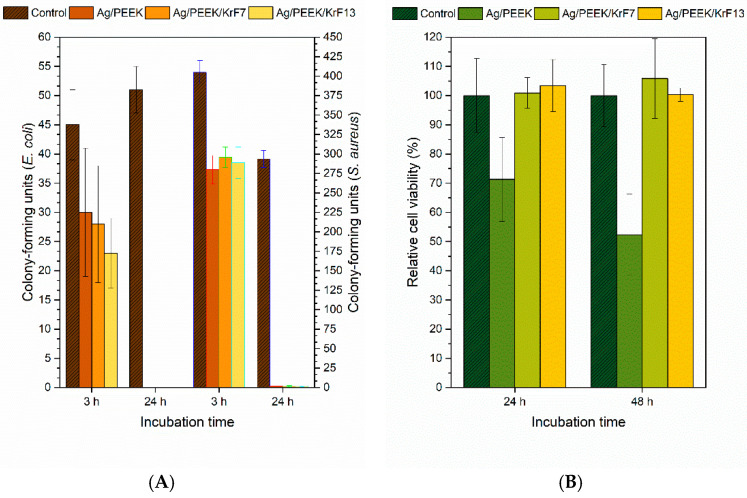
Results of biological tests showing (**A**) data from drop plate tests performed against *Escherichia coli* (left Y-axis) and *Staphylococcus aureus* (right Y-axis) after 3 and 24 h of incubation, expressed as the number of CFU, and (**B**) non-cytotoxic effects of Ag/PEEK/KrF7 and Ag/PEEK/KrF13 samples on primary lung fibroblasts cultivated for 24 and 48 h, expressed as relative cell viability (%).

Because we were interested in the antibacterial properties of prepared composites, we performed drop plate tests on Ag/PEEK, Ag/PEEK/KrF7, and Ag/PEEK/KrF13 due to their different concentrations of silver. In this work, tests were performed on *Escherichia coli* and *Staphylococcus aureus*, one of the most common strains of bacteria, which cause catheter-associated urinary tract infections (CAUTI) and are responsible for most medical device-related infections [38,39,40]. The antibacterial activity results are shown in Figure 7A. After 3 h of incubation time, the samples did not show antibacterial activity against both strains of bacteria. However, the CFU of *E. coli* decreased slightly after interacting with all the samples containing silver. A significant decrease occurred in the case of Ag/PEEK/KrF13, which may have been caused by the combination of silver nanoparticles and the structural surface morphology. Similar trends were reported by Kaimlová et al. [41] in their work on silver nanowires supported by laser-patterned polyethylennaphthalate. More interesting results occurred after 24 h of incubation time. The antibacterial effect was revealed for all Ag-doped samples against both strains of bacteria. Antibacterial effects on *E. coli* and *S. aureus* were also observed in the study by Deng et al. [20] where PEEK was used as a 3D-printed implant with AgNPs. Furthermore, total inhibition of bacterial growth was crucial in the case of *S. aureus*, which is known for its antibiotic resistance [42]. The antibacterial properties of Ag/PEEK/KrF7 and Ag/PEEK/KrF13 were also presumably seen due to the release of Ag^+^ ions as the surface morphology and chemical composition were similar to those of the laser pre-treated samples in our previous work [8]. Our previous work dealt with the study of the long-term release of Ag^+^ ions from KrF-laser-pre-treated PEEK with immobilised AgNPs, which exhibited an antibacterial effect against *E. coli* even though the concentration of Ag^+^ ions was approximately 40 µg·L^−1^ under static conditions after 24 h of leaching. Furthermore, Ning et al. reported the MIC of Ag^+^ ions for *E. coli* and *S. aureus* to be approximately 27 µg·L^−1^ [43].

### 3.3. Cytotoxicity Tests

As prepared composites are designed for biomedical applications, the cytotoxic effects of Ag/PEEK, Ag/PEEK/KrF7, and Ag/PEEK/KrF13 were tested on primary lung fibroblasts. The results of these tests are presented in Figure 7B. From the graph, it is obvious that Ag/PEEK showed weak toxicity after 24 h of incubation time. A moderate cytotoxic effect was observed after a longer incubation time, which is alarming; therefore, Ag/PEEK is not a suitable candidate for biomedical applications. Cytotoxicity occurred due to the high concentration of silver nanoparticles and the low surface roughness, as it is known that surface-modified PEEK is not toxic in relation to cell adhesion and proliferation [26]. However, this composite can be used as a support material for the packaging of medical devices with poor sterilisation or storage conditions. The most significant observation emerging from these tests was the non-cytotoxic effect of the samples after modification with the KrF laser after 24 and 48 h of incubation time. Cells adhered and proliferated on the surfaces of Ag/PEEK/KrF7 and Ag/PEEK/KrF13 due to the various surface morphologies because surface morphology and roughness are important for cell adaptation [44]. Not only does surface morphology play a role in cell adhesion and proliferation but it also affects the form and concentration of silver. In the case of Ag/PEEK, AgNPs are distributed all over the surface of PEEK, so cells practically come into contact with the silver layer, which is toxic to them. After KrF laser modification, the nanostructures occurred and the concentration of silver decreased, offering more appropriate conditions for cell adhesion. Similar cytotoxicity tests were performed on polyethylennaphthalate (PEN) and also LIPSS decorated with silver nanowires (AgNWs) [45]. Unfortunately, the cytotoxicity tests showed strong cytotoxicity in the case of AgNWs/PEN even after 72 h of incubation time.

## 4. Conclusions

In summary, the investigation into polyetheretherketone (PEEK) with immobilised silver nanoparticles (AgNPs) has unveiled a promising avenue for advanced material engineering. Examination of LIPSS induced by KrF laser treatment demonstrates the potential for precise surface modifications with substantial implications for improving material properties. Moreover, these structures can be explored as a basis for direction control grown from specific cells (myocytes or bone cells). The mass transfer effects observed after GaN laser treatment offer a novel avenue for controlled material manipulation. Although plasma treatment resulted in non-significant changes in surface morphology, this finding underlines the nuanced nature of material modifications and the need for further investigation and optimisation. The XPS and UV-Vis analyses revealed stability of the surface chemistry and optical activity of KrF-laser-treated composites in contrast to plasma-modified. The discovery of the complete inhibition of *Escherichia coli* and *Staphylococcus aureus*, without cytotoxic effects, highlights the promise of these materials in medical and antibacterial applications. Collectively, these findings suggest that these developments in material science hold significant potential for various medical applications, making PEEK an attractive candidate for medical and healthcare applications where the complete inhibition of *E. coli* and *S. aureus* has significant implications. Further research and exploration in these areas promise to bring about even more exciting possibilities for the future.

## Figures and Tables

**Table 1:** Surface parameters such as mean surface roughness (*R*_a_), surface area difference (SAD), and periodicity (*L*) of selected samples.

Sample	*R*_a_ (nm)	SAD (%)	*L* (nm)
PEEK	2.4	-	-
Ag/PEEK	3.6	4.6	-
Ag/PEEK/KrF7	31.3	52.7	210 ± 8
Ag/PEEK/KrF13	28.6	34.9	223 ± 4
Ag/PEEK/GaN60	6.8	2.3	-
Ag/PEEK/GaN240	3.1	1.1	-

## Data Availability

The data presented in this study are available at https://doi.org/10.5281/zenodo.10234756, accessed on 27 November 2023.
